# Is more better? An analysis of toxicity and response outcomes from dose-finding clinical trials in cancer

**DOI:** 10.1186/s12885-021-08440-0

**Published:** 2021-07-05

**Authors:** Kristian Brock, Victoria Homer, Gurjinder Soul, Claire Potter, Cody Chiuzan, Shing Lee

**Affiliations:** 1 Cancer Research UK Clinical Trials Unit, University of Birmingham, Birmingham, UK; 2 Institute of Cancer and Genomic Sciences, University of Birmingham, Birmingham, UK; 3 Mailman School of Public Health, Columbia University, New York, NY USA

**Keywords:** Dose finding, Phase I, Toxicity, Efficacy, Response, Monotone, Cancer

## Abstract

**Background:**

The overwhelming majority of dose-escalation clinical trials use methods that seek a maximum tolerable dose, including rule-based methods like the 3+3, and model-based methods like CRM and EWOC. These methods assume that the incidences of efficacy and toxicity always increase as dose is increased. This assumption is widely accepted with cytotoxic therapies. In recent decades, however, the search for novel cancer treatments has broadened, increasingly focusing on inhibitors and antibodies. The rationale that higher doses are always associated with superior efficacy is less clear for these types of therapies.

**Methods:**

We extracted dose-level efficacy and toxicity outcomes from 115 manuscripts reporting dose-finding clinical trials in cancer between 2008 and 2014. We analysed the outcomes from each manuscript using flexible non-linear regression models to investigate the evidence supporting the monotonic efficacy and toxicity assumptions.

**Results:**

We found that the monotonic toxicity assumption was well-supported across most treatment classes and disease areas. In contrast, we found very little evidence supporting the monotonic efficacy assumption.

**Conclusions:**

Our conclusion is that dose-escalation trials routinely use methods whose assumptions are violated by the outcomes observed. As a consequence, dose-finding trials risk recommending unjustifiably high doses that may be harmful to patients. We recommend that trialists consider experimental designs that allow toxicity and efficacy outcomes to jointly determine the doses given to patients and recommended for further study.

**Supplementary Information:**

The online version contains supplementary material available at (10.1186/s12885-021-08440-0).

## Background

A goal of dose-finding clinical trials is to evaluate outcomes under a set of investigational doses. A common general approach starts by giving a relatively low dose to a small cohort of patients. The outcomes of this cohort affect the dose given to the next. For instance, if no unacceptable toxic reactions are seen in this initial cohort, the next cohort is likely to be given a higher dose. However, if outcomes are adverse, the next cohort might be given the same dose or a lower dose, or the trial might be halted altogether. This sequential and adaptive approach continues until the experimental design identifies a suitable dose. This pattern of starting low and seeking to escalate dose justifies the descriptor *dose-escalation trials*.

The most common approach [1, 2] in dose-escalation trials is the family of rule-based A+B designs, the most famous example of which is the perennial 3+3 design [3]. It evaluates doses in cohorts of three patients, using a set of rules to escalate dose so long as an unacceptably high incidence of dose-limiting toxicity (DLT) is not seen.

The main alternative class of dose-escalation methodology comprises the so-called model-based designs. These use statistical models to estimate the dose-event curve. Whilst model-based methods are used far less frequently than rule-based methods, two designs that have seen relatively wide use in recent years [2] are the continual reassessment method (CRM) [4] and the escalation with overdose control (EWOC) method [5].

Biostatisticians have encouraged trialists to shift from rule-based to model-based methods, largely because they offer better performance [6–8]. The two approaches, however, share some fundamental assumptions. Firstly, they each assume that the probability of DLT increases as dose is increased. This reflects the toxicologists’ adage *the dose makes the poison*, a rule that is generally accepted without challenge. The 3+3, CRM and EWOC designs all select doses based only on binary DLT outcomes in pursuit of the maximum tolerable dose (MTD), the highest dose with toxicity probability less than some critical pre-specified value. So long as two doses are deemed tolerable, the higher dose is favoured. Efficacy outcomes like response or survival are not formally used in dose-selection decisions. There are variants of the CRM design [9, 10] and other statistical approaches [11–13] for dose-finding by co-primary toxicity and efficacy outcomes. However, these have experienced comparatively little use [2, 14].

In dose-finding trials, investigators seek to identify tolerable and efficacious doses. The reliance solely on a toxicity outcome in the majority of dose-finding trials dictates that an assumption is made about the efficacy outcome.

*The assumption is that higher doses are always more efficacious*.

We refer to this as the monotonic efficacy assumption. In MTD-seeking trials, investigators will escalate dose without formal reference to an efficacy outcome. When the monotonic efficacy assumption holds, the MTD maximises the expectation of efficacy for a specified risk of toxicity. However, a plausible way in which the monotonic efficacy assumption can be violated is when the probability of efficacy plateaus at some critical dose. Escalation beyond this point exposes patients to a greater risk of toxicity for no accompanying increase in the probability of efficacy. As such, the monotonic efficacy assumption is rather more controversial than the monotonic toxicity assumption.

Monotonic efficacy has a plausible rationale in the treatments that have formed the backbone of anti-cancer therapies for decades. Cytotoxic treatments like chemotherapy damage tumours and healthy tissue alike. The presence of toxicity is a sign that anti-tumour activity is probably happening as well. In this setting where toxicity and efficacy are broadly accepted to be coincident, the use of dose escalation designs became commonplace. In recent years, however, numerous targeted therapies, immunotherapies and cell therapies have been approved for use against cancer. In a recent systematic review of dose-finding methodologies used between 2008 and 2014, Chiuzan et al. [2] found that over half of the trials investigated a targeted therapy or immunotherapy and the overwhelming majority used an MTD-seeking method. With these novel treatment classes, the rationale for assuming that efficacy always increases in dose is less clear.

There are notable instances in the literature where a monotonic dose-efficacy relationship has not been observed. For instance, a major recent success in the development of novel anti-cancer drugs has been the PD-1 blockade antibody, pembrolizumab. A phase I trial investigated pembrolizumab doses of 1 mg/kg, 3 mg/kg and 10 mg/kg every 2 weeks, and 2 mg/kg and 10 mg/kg every 3 weeks in 30 patients with various malignancies [15]. Large expansion cohorts in non-small-cell lung cancer (NSCLC) further evaluated 495 patients at doses 2 mg/kg or 10 mg/kg every 3 weeks or 10 mg/kg every 2 weeks [16]. Subsequently, a phase III trial that contributed to a licensing application in NSCLC randomised 345 patients to pembrolizumab 2 mg/kg, 346 to pembrolizumab 10 mg/kg, and compared each of these experimental arms to a control arm comprising 343 patients randomised to docetaxel [17]. Despite the wide range of doses investigated in large sample sizes, the phase III trial observed very similar overall survival and RECIST response outcomes in the two pembrolizumab doses, with each producing materially better outcomes than the control arm. The drug was subsequently licensed at 200 mg (i.e. not adjusted for patient weight) every 3 weeks, reflecting the absence of extra efficacy at higher doses. Assuming an average adult weight of 70-80kg, the licensed dose is situated at the lower end of the doses investigated throughout these three clinical trials.

We naturally wonder about the suitability of the monotonic efficacy assumption in a wider sense. In this manuscript, we investigate two related questions. What evidence is there that the probabilities of a) toxicity, and b) efficacy increase in dose in modern cancer therapies?

## Methods

We sought to identify a broad sample of manuscripts reporting recent dose-finding clinical trials in oncology.

### Identifying manuscripts

Chiuzan et al. [2] conducted a systematic review of the methods used in cancer dose-finding trials. Their findings mirrored those of Rogatko et al. [1] from the previous decade that over 90% of dose-finding trials use a rule-based design like 3+3. The authors found 1,712 manuscripts published between 2008 and the first half of 2014. The authors published in their paper a large table summarising the trials that used model-based methods, like CRM or EWOC, of which there were 92 examples. Whilst extracting data from 1,712 papers would be infeasible, extracting data from 92 was possible. However, the subset of trials that use model-based methods may not be representative of the entire sample. For this reason, we supplemented the list of 92 model-based papers with 30 randomly-selected papers that used rule-based methods, stratified by year of publication. Combined, this produced a sample of 122 manuscripts.

### Extracting data

Each of the 122 manuscripts [18–139] presented the results of at least one dose-finding experiment in humans. Some papers reported the results of more than one experiment. From each experiment, we extracted descriptive data pertaining to the patient population, the dose-varying treatment, and any concomitant treatments. Concerning outcomes, we extracted the dose-levels administered, the number of patients evaluated at each, and the number of DLT and objective response events recorded at each. These outcomes are explained and justified in the following sections.

#### Dose-level outcomes vs pooled outcomes

We only recorded outcomes broken down by dose-level because these would allow us to address the research question pertaining to the evidence for monotonically increasing toxicity and efficacy probabilities. We did not collect outcomes that were reported by pooling all dose-levels because this would not address our research question.

#### Toxicity outcomes

Dose-limiting toxicity (DLT) is the de-facto standard safety outcome in dose-finding trials. Manifestation of DLT involves the occurrence of pre-specified adverse events (AEs) that are serious enough that they would motivate the clinicians to rule out higher doses in the affected patient or consider the temporary suspension or complete cessation of current therapy. There is no standard definition of DLT across trials but the outcome would be defined in each trial protocol and remain consistent across the doses investigated within each trial. The definition of DLT in a given trial may reflect the clinical characteristics of the disease and the anticipated adverse events from the entire treatment ensemble (i.e. arising from the experimental therapy and concomitant therapies).

Data on DLT outcomes were sought in every manuscript. We analyse outcomes for DLT because it was the most widely-reported toxicity outcome measure.

#### Efficacy outcomes

The question motivating this research concerns drug efficacy and how this changes as dose is increased. *Efficacy* is only loosely defined in cancer. There is no single outcome that is unambiguously accepted as the variable best reflecting efficacy. Applications for drug licensing are generally supported by phase III trials that use survival outcomes like overall survival (OS) and progression-free survival (PFS). In contrast, early phase trials, when they evaluate efficacy, tend to use surrogate outcomes that can be evaluated over the short-term like disease response.

Assessing disease response generally involves comparing the extent of disease (e.g. tumour size or number of leukaemic cells) at baseline and after treatment administration to characterise the patient’s response to treatment using one of several categories. RECIST [140] is the most common response outcome categorisation used in solid tumour trials. RECIST categorises each disease assessment as one of: complete response (CR); partial response (PR); stable disease (SD); or progressive disease (PD).

Researchers have defined analogues to RECIST in other cancers, including blood cancers where diseased cells reside in the blood rather than a discrete measurable mass. An example of this is the Cheson criteria in acute myeloid leukaemia (AML) [141] and iwCLL criteria in chronic myeloid leukaemia [142]. These contain response categories that are similar to those in RECIST, with slight modifications to reflect the phenomena specific to the disease.

Under RECIST, an objective response (OR) is said to occur when a patient experiences CR or PR. Under the RECIST analogues, further response categories are included in OR. For instance, in AML, a patient with complete remission with incomplete blood count recovery would be considered to have experienced OR.

Data on OR outcomes were sought in every manuscript. We analyse outcomes for OR because it was the most widely-reported efficacy outcome measure.

#### Orderability of doses

Analysing how the probabilities of events change as dose increases requires that we are working with increasing doses. The general 3+3, CRM and EWOC methods require that the doses under investigation are *fully orderable*. That is, we need to be able to unambiguously say that *d*_*i*_<*d*_*j*_ or *d*_*i*_>*d*_*j*_ for each pair of doses in the set of doses under investigation.

When we encountered dose-levels that were not fully orderable, for the purposes of conducting statistical analysis we broke the doses up to form fully orderable subsets that we called *analysis series*.

There are many possible subsets of a set so the way we formed the analysis series was unavoidably subjective. To promote objectivity, we followed some simple rules. We sought to maximise the size of the largest fully orderable series. Furthermore, we avoided allocating a dose to several series unless repetition was the only way to avoid having an orphan dose (i.e. a series of size 1).

Consider, for instance, the three dose scenario: dose 1 = 10mg of drug A + 20mg of drug B; dose 2 = 20mg A + 10mg B; dose 3 = 20mg A + 20mg B. This set of doses is not totally orderable because it is impossible to say whether dose 1 is higher or lower than dose 2. However, each of these doses is categorically less than dose 3. Thus, in this hypothetical scenario, we would have analysed outcomes of the two totally orderable subsets (dose 1, dose 3) and (dose 2, dose 3). In doing so, the outcomes at dose 3 would have featured in the analyses twice. The alternative would have been to arbitrarily throw away the outcomes at dose 1 or dose 2, an option we rejected because it is wasteful.

In summary, the data have been recorded in a way amenable to answering the research questions.

#### Database creation

Data were extracted from papers and recorded on prior-written standardised forms. The data were then recorded on sheets in an Excel file that was deposited in the University of Birmingham’s data repository [143].

Data were extracted by VH, GS, KB and CP. Data for 52 manuscripts were extracted by two different authors and differences were resolved by discussion. Data for 70 manuscripts were extracted by one author.

### Analysis model

The DLT and OR outcomes we extracted were binary in nature. Outcomes were analysed within study using Emax models [144].

Emax is a flexible non-linear approach for fitting sigmoidal (i.e. S-shaped) dose-response curves to continuous or binary responses. In our analysis, the binary response variable was the patient-level presence of DLT or OR. The explanatory variable was the dose-level administered. Emax can capture relationships where the mean response increases in dose, decreases in dose, or is independent of dose. Separate models were fit to the collection of patient outcomes in each study. Outcomes from different studies were not pooled because of the disparate patient populations and definitions of DLT and OR. Each of these factors remained consistent within each analysis series. The fitted values from the Emax models represent the event probabilities at each dose, ranging from 0 to 1.

Binary outcomes might generally suggest analysis via logit linear regression models. We did not use logit models because they assume that the event probability invariably tends to 1 with large enough predictor values. This is inappropriate in our analysis that seeks to investigate how event probabilities vary with dose, including the possibility that event probabilities plateau at values less than 1. Whilst it may be acceptable to assume that a high enough dose will guarantee a toxic outcome, empirically it is inappropriate to assume that a high enough dose guarantees an efficacious response. The relative strength of the Emax model is that it allows the event probability to plateau at a value less than 1. It contains as a special case the scenario reflected by logit models where the event probability tends to 1 as dose is increased.

We used both maximum likelihood and Bayesian approaches to fit Emax models. Maximum likelihood models were fit because they do not require the specification of priors. However, the analysis series in this research were very small, with some data-sets including fewer than 10 patients. In several instances, maximum likelihood models failed to converge. In these circumstances, Bayesian models can be extremely valuable because the specification of very small amounts of information in prior distributions promotes model convergence. Bayesian models succeeded in estimating dose-event curves in all instances. Our chosen prior distributions are introduced briefly below and expanded in detail in the supplementary appendix.

The height of each dose-event curve was calculated as the model-fitted event probability at the highest dose under investigation minus that of the lowest dose. This concept is illustrated graphically in Figure 1 in the supplementary appendix.

#### Prior distributions

For the Bayesian analyses, we were required to specify prior distributions on the four parameters in the Emax model. We selected uniform priors on the parameters that reflect the minimum and maximum event probabilities, constrained to take values only in the region from 0 to 1. This meant that all event probabilities at the lowest and highest doses were equally likely, with no inclination towards a particular probability.

Prior distributions on the other two parameters, that control the location and steepness of the S-curve, were chosen to provide very modest information to constrain estimation to the region of feasible values. Full details on prior selection are given in the supplementary appendix, including in supplementary Figure 2 a plot of candidate event curves that are generated by the priors.

#### Appraising model fits

Model-generated dose-event curves were inspected visually alongside source data to verify the quality of model fits. Furthermore, we recorded metrics for all Bayesian models that can diagnose a potentially poor model fit. Further details are given in the supplementary appendix.

#### Software

Maximum-likelihood Emax models were fit using the DoseFinding package [145] and Bayesian models were fit using the brms package [146] in R [147]. Data processing was aided using tidyverse [148] packages, posterior samples were extracted using tidybayes [149], and plots were produced using ggplot2 [150].

## Results

**Fig. 1:**
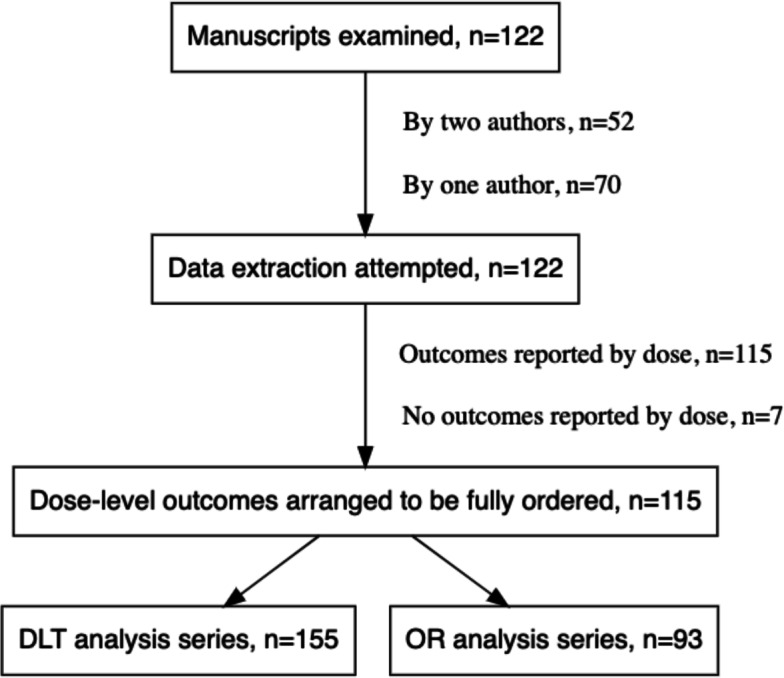
The data extraction process. The procedure for forming fully orderable subsets of doses is described in the text

115 (94%) of the 122 examined manuscripts reported outcomes by dose-level. After extracting data and creating fully-orderable dose subsets, these yielded 155 analysis series for DLT, and 93 analysis series for OR (Fig. 1). Characteristic information is summarised in Table 1.

**Table 1:** Characteristics of manuscripts studied and outcome series extracted

	Manuscripts, N = 115	DLT series, N = 155	OR series, N = 93
**Year of publication**			
2008	13 (11%)	17 (11%)	13 (14%)
2009	9 (7.8%)	14 (9.0%)	10 (11%)
2010	9 (7.8%)	10 (6.5%)	8 (8.6%)
2011	20 (17%)	23 (15%)	11 (12%)
2012	26 (23%)	35 (23%)	23 (25%)
2013	16 (14%)	22 (14%)	16 (17%)
2014	22 (19%)	34 (22%)	12 (13%)
**Experimental design**			
Model-based	85 (74%)	116 (75%)	56 (60%)
Rule-based	30 (26%)	39 (25%)	37 (40%)
**Disease class**			
Non-haematological	82 (71%)	117 (75%)	71 (76%)
Haematological	30 (26%)	31 (20%)	18 (19%)
Both	2 (1.7%)	6 (3.9%)	4 (4.3%)
Not disclosed	1 (0.9%)	1 (0.6%)	0 (0%)
**Disease**			
Solid tumours		48 (31%)	29 (31%)
Breast cancer		9 (5.8%)	8 (8.6%)
Gastrointestinal cancer		10 (6.5%)	6 (6.5%)
AML		7 (4.5%)	6 (6.5%)
Lung cancer		9 (5.8%)	3 (3.2%)
Lymphoma		6 (3.9%)	5 (5.4%)
Multiple myeloma		6 (3.9%)	4 (4.3%)
Glioma		5 (3.2%)	4 (4.3%)
Melanoma		4 (2.6%)	4 (4.3%)
Mixed haematological cancers		8 (5.2%)	0 (0%)
CNS tumours		4 (2.6%)	3 (3.2%)
Head and neck cancer		4 (2.6%)	3 (3.2%)
Brain cancer		5 (3.2%)	1 (1.1%)
Lymphoma and advanced solid tumours		4 (2.6%)	2 (2.2%)
Sarcoma		3 (1.9%)	3 (3.2%)
CLL		3 (1.9%)	2 (2.2%)
Hepatocellular carcinoma		2 (1.3%)	2 (2.2%)
Renal cell carcinoma		2 (1.3%)	2 (2.2%)
Biliary tract cancer		3 (1.9%)	0 (0%)
Glioblastoma		2 (1.3%)	1 (1.1%)
NSCLC		2 (1.3%)	1 (1.1%)
SCLC		3 (1.9%)	0 (0%)
Cervical cancer		1 (0.6%)	1 (1.1%)

**Table 1:** Characteristics of manuscripts studied and outcome series extracted (*Continued*)

	Manuscripts, N = 115	DLT series, N = 155	OR series, N = 93
Colorectal cancer		1 (0.6%)	1 (1.1%)
Gynaecological cancer		1 (0.6%)	1 (1.1%)
B-cell malignancies		0 (0%)	1 (1.1%)
Not disclosed		1 (0.6%)	0 (0%)
Pancreatic cancer		1 (0.6%)	0 (0%)
Rectal cancer		1 (0.6%)	0 (0%)
**Treatment undergoing dose-escalation**			
Inhibitor		58 (37%)	43 (46%)
Chemotherapy		51 (33%)	25 (27%)
Chemotherapy + inhibitor		17 (11%)	12 (13%)
Monoclonal antibody		8 (5.2%)	0 (0.0%)
Immunomodulatory		4 (2.6%)	2 (2.2%)
Immunomodulatory + chemotherapy		3 (1.9%)	2 (2.2%)
Radiotherapy		4 (2.6%)	1 (1.1%)
Oncolytic virus		2 (1.3%)	2 (2.2%)
Radiopharmaceutical + inhibitor		2 (1.3%)	2 (2.2%)
Cell therapy		1 (0.6%)	2 (2.2%)
Antibody-drug conjugate		1 (0.6%)	1 (1.1%)
Cytokine		1 (0.6%)	1 (1.1%)
Chemoprevention		1 (0.6%)	0 (0%)
Gene therapy		1 (0.6%)	0 (0%)
Not disclosed		1 (0.6%)	0 (0%)
Treatment ensemble contains chemotherapy		91 (59%)	49 (53%)
Number of dose-levels		4 (2, 5)	4 (2, 5)

### Patient groups

Three-quarters of the data analysed came from non-haematological cancers. In approximately one third of cases, the patient group comprised different types of solid tumour. Most commonly, however, trials were conducted within specific cancer sites, with breast, gastrointestinal, and lung cancers featuring relatively frequently.

Approximately one quarter of the data came from trials in haematological malignancies. Once again, trials were sometimes conducted in fairly heterogeneous patient groups and sometimes in specific diseases like AML, CLL and lymphoma.

### Experimental treatments

The treatment type most commonly undergoing dose-escalation was inhibitors, contributing 58 (37%) DLT series and 43 (46%) OR series. Chemotherapies were also common targets for dose-escalation, contributing 51 (33%) DLT series and 25 (27%) OR series. Monoclonal antibodies were fairly infrequent in this data set, contributing only 8 (5%) DLT series and zero OR series. Trials that escalated dose in two different treatment types were common, with chemotherapy plus inhibitor the most common pairing, yielding 11% of the DLT series and 13% of the OR series. The median of doses in an analysis series was 4 (IQR = 2, 5).

### Monotonicity of DLT and OR in dose

**Fig. 2:**
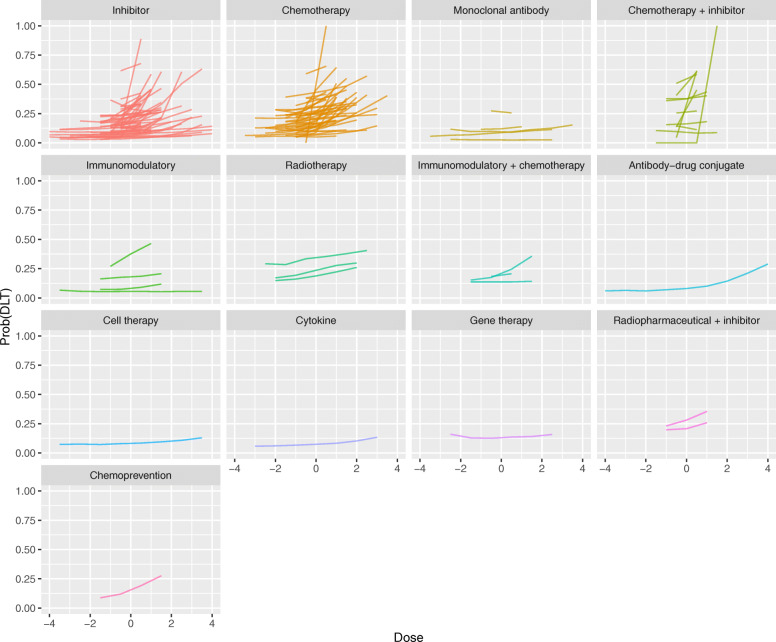
Fitted dose-DLT curves yielded by Bayesian Emax models. For presentation, doses are centralised at zero (i.e. the average dose-level for each series is subtracted) and up to the middle nine doses are shown, to allow the series to be visualised together

Fitted curves for the dose-DLT series produced by the Bayesian Emax models are shown in Fig. 2. Each line reflects the best fit to all of the DLT outcomes observed in one analysis series. A separate panel is shown for each type of treatment that underwent dose-escalation. Information on patient group and concomitant therapies are not shown in this plot.

We see that the majority of the fitted DLT series show a positive relationship with dose, reflecting that DLT becomes more likely as dose is increased. This is seen in all types of therapy and matches our expectation that the dose makes the poison. The fitted series for inhibitors and chemotherapies appear to increase more steeply than for other therapies.

**Fig. 3:**
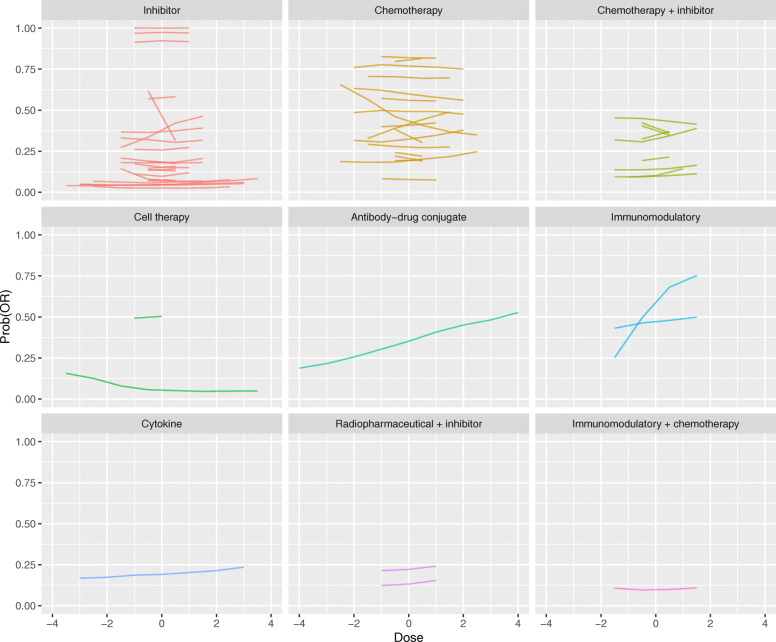
Fitted dose-OR curves yielded by Bayesian Emax models. For presentation, doses are centralised at zero (i.e. the average dose-level for each series is subtracted) and up to the middle nine doses are shown, to allow the series to be visualised together

Fitted curves for the dose-OR series produced by the Bayesian Emax models are shown in Fig. 3. Contrasting to Fig. 2, we see that there are materially fewer OR curves than DLT curves. However, it is clear that the fitted OR curves are much less likely to show a strong positive association between dose and response. It is striking how few positive gradients there are. Even amongst chemotherapies, there is scant evidence of greater efficacy at higher doses. The single OR series for an antibody-drug conjugate and one of the series for an immunomodulatory drug show comparatively strong evidence of a positive dose-response effect.

**Fig. 4:**
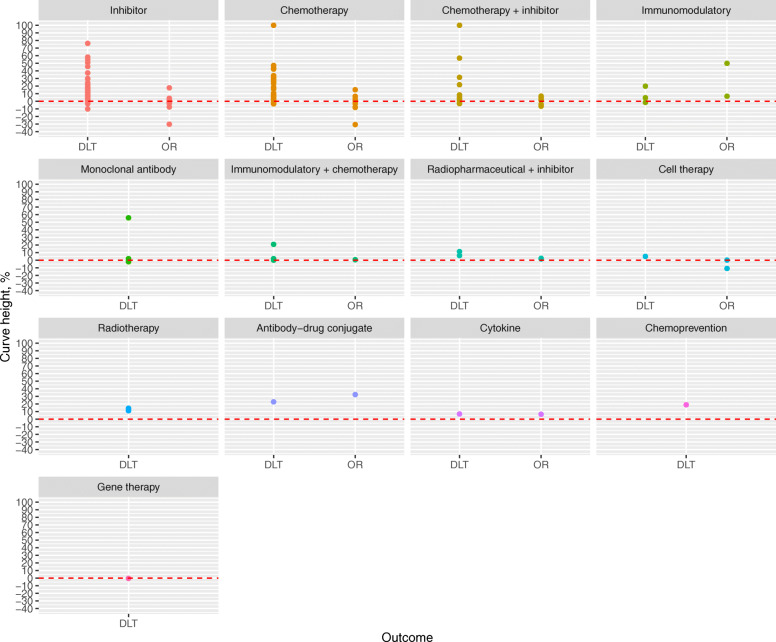
Heights of dose-DLT and dose-OR curves. The dashed red lines reflect a curve height of zero where there is no association between dose and event. Positive values reflect that event probabilities increase as dose is increased, and negative values reflect a decreasing probability as dose is increased

The heights of the fitted dose-DLT and dose-OR curves are shown in Fig. 4, with statistics for the two outcomes plotted side-by-side within treatment type. The dashed red line at zero reflects the null value where there is no relationship between dose and event. Positive curve heights indicate that the event became more likely as dose is increased, and vice-versa.

We see that the majority of DLT curves show a positive curve height in all classes of treatment. There are infrequent series that suggest no relationship, or a modestly negative relationship. In stark contrast, the same statistics for the OR series straddle the null line in the most frequent treatment categories, demonstrating a lack of evidence in support of the monotonic efficacy assumption.

Furthermore, the curve heights of the DLT series show much greater variability than those of the OR series. There are some instances where the probability of DLT increased quite rapidly as dose increased.

Curve heights for DLT and OR series are plotted by type of disease in Figure 3 in the supplementary appendix. We see that the phenomena we have described are broadly observed across most disease types.

Similarly, Figure 4 in the supplementary appendix shows the equivalent inferences split by class of dose-finding methodology (i.e. rule-based or model-based). The two OR series with the steepest positive relationship with dose both use a rule-based design. Generally, however, the heights of the DLT and OR curves from studies that used model-based designs were similar to those yielded by rule-based designs. In both groups, the observation remained that DLT curves commonly increased with dose whilst OR curves were mostly invariant in dose.

The inferences thus far have come from the Bayesian Emax models, where the statistical model fitting procedure succeeded in all instances. We also sought to analyse series using maximum likelihood Emax models. The maximum likelihood model fitting procedure failed in many instances. Examples of model-fitting failure were procedures that did not converge, or procedures that yielded extremely large estimates for the standard errors of model parameters. For completeness, we show the fitted DLT and OR series yielded by the maximum likelihood models in Figures 5 and 6 in the supplementary appendix. The fitted series produced by the maximum likelihood models are less smooth. Supplementary Figure 7 shows that the curve heights estimated by maximum likelihood models are generally more extreme than those produced by Bayesian models, with more values positive and negative values far from zero for DLT and OR series. In the most frequently investigated treatments, once again we see that the DLT series are overwhelmingly likely to have a positive curve height, whilst the heights of OR series cluster around zero.

## Discussion

We collected dose-level toxicity and efficacy outcomes from 115 papers reporting early phase clinical trials of experimental anti-cancer therapies. These trials all used experimental methods that assume that higher doses are associated with greater probabilities of both toxicity and efficacy. We then analysed those outcomes using flexible non-linear regression methods. In summary, we found broad evidence that the probability of toxicity increased in dose in most treatment classes, in most types of cancer, in scenarios that use rule-based or model-based dose-escalation methodologies. In contrast, we found very little evidence that the probability of response increased as dose was increased.

On the face of it, the implication of our findings is that dose-escalation clinical trials commonly advocate doses that are unjustifiably high. For a treatment where toxicity incidence is positively associated with dose and response incidence is invariant to dose, lower doses should be preferred. However, by conducting dose-escalation experiments that recommend doses only by toxicity outcomes, explicitly assuming that higher doses are more efficacious and therefore preferable, many trials miss the opportunity to recommend a lower dose with less toxic effects and no commensurate cost to efficacy. This could help explain why dose-reduction occurs in phase II and III clinical trials. For example, a detailed account of post-phase I dose optimisation in an inhibitor drug is given by Lee et al. [151].

The statements above pertain to the ranges of doses investigated in phase I clinical trials. They do not apply to the ranges of all possible doses. Naturally, we acknowledge in all active therapies that there must be a dose so low that the attendant anti-tumour effect is negligible. That we found many series with non-trivial response rates at all doses is perhaps testament to the value of pre-clinical research and PK/PD modelling in locating dose ranges that are likely to be tolerable and active for phase I trials.

A logical remedy to the problems we have described would be to use so-called seamless phase I/II designs that recommend doses by toxicity and response outcomes, several examples of which have been published [9–13] and implemented in clinical trials [14, 152]. These designs bring their own challenges, the most notable of which are the extra statistical complexity and the requirement that the co-primary outcomes can be evaluated over a similar time horizon to allow dose-escalation decisions to be made in a timely manner.

A simpler solution would require early phase trialists to address the assumptions made by their phase I designs and discuss the appropriateness of their final dose recommendations in light of toxicity and efficacy outcomes. By reporting suitable efficacy and toxicity outcomes by dose, researchers would allow the community to assess the suitability of the methodological assumptions and identify doses most appropriate for further study. Arguably, this already occurs in practice when trialists use dose-escalation methods like 3+3, CRM or EWOC cognisant of the possibility that higher doses might not bring greater efficacy. The drawback of this putative approach is that it risks allocating patients in the dose-finding trial to inappropriate doses. Trialists that regard this as unethical would be encouraged to use seamless phase I/II methodologies, described above.

It is possible that monotonic efficacy effects in dose are observed in long-term clinical outcomes like OS and PFS, even if they are not seen in surrogate outcomes like response. We cannot provide any evidence to support or refute this hypothesis since survival outcomes were reported by dose extremely infrequently. If this hypothesis is true, however, it calls into question the validity of objective response as a surrogate outcome for clinical outcomes.

The sample size of the trials included in this research is small. Researchers might legitimately ponder the feasibility of detecting strong dose-event relationships with such small sample sizes. It was advantageous, then, that we included and analysed DLT outcomes because they have shown that evidence of stark relationships can be garnered from small trials, particularly when analysed together. Within study, OR outcomes routinely failed to show the strength of relationship with dose shown by DLT outcomes.

It is perhaps self-fulfilling that we have observed stark monotonic effects in toxicity because dose-finding trials escalate dose in the absence of unacceptable toxicity and de-escalate dose or halt the trial when toxicity manifests. For this reason, it is plausible that the toxicity relationships we have presented here may be biased upwards. Nevertheless, this does not explain the lack of evidence for a relationship between dose and efficacy.

Recently, Hazim et al. [153] investigated the relationship between dose and efficacy in single-agent phase I trials in oncology. They did so by calculating aggregate response rates at dose categories defined by the ratio of the given dose to the recommended phase II dose (RP2D). They found evidence that response rates increased modestly as doses approached the RP2D. Compared to their study, this research benefits from including treatment combinations, including both efficacy and toxicity outcomes, and using statistical modelling.

### How could dose-finding be conducted in modern cancer therapeutics?

We conclude this article with some discussion on the design, conduct and reporting of dose-finding clinical trials.

It is our opinion that early phase clinical trials of modern cancer therapies should seek to optimise the delivery of treatments, balancing the risk of harmful toxicity with the expectation of improving meaningful clinical outcomes. This same sentiment was recently advocated by early phase trial methodologists at MD Anderson [154]. Naturally, this requires that toxicity and efficacy outcomes are evaluated, and used to adapt the doses given to future patients.

Investigators should be clear on what constitutes a meaningful clinical outcome. OS and quality of life measures are widely seen as gold standard outcomes for demonstrating benefit to patients and, in many settings, PFS is also considered a valid clinical outcome. Overwhelmingly, however, we have seen that efficacy is measured in dose-finding trials only by categorical response outcomes.

Where response has been validated as a reliable surrogate for a clinical outcome in a patient group, it should continue to be used to measure efficacy in dose-finding trials. In this scenario, we recommend using co-primary toxicity and response outcomes in a phase I/II dose-finding design. These designs are unified by the belief that the MTD may not be the best dose. Many examples of such designs have been published [9–13] and more will surely be added. More recently, methods have been developed for modeling toxicity and time to event outcomes like PFS [155, 156].

Comparing to traditional phase I designs that only assess toxicity, seamless phase I/II designs will likely take longer to run and require more patients. However, as phase I/II designs also answer the question typically investigated in a traditional phase II trial, the more appropriate comparison is the total time and sample size required to run a phase I followed by a phase II trial. By this comparison, when considering that a seamless design requires only one protocol and no break between trials, a seamless design may not require more resource than separate trials.

Where response has not been validated as a reliable surrogate for a clinical outcome in a patient group, the situation is much more challenging. If investigators seek to avoid using a surrogate of unknown utility, the entire clinical trial program will amount to contrasting clinically meaningful outcomes at several different doses, perhaps compared to a shared control, incorporating pre-specified decisions to narrow the experimental doses, culminating in a final test of superiority. These goals can be achieved using the multi-arm multi-stage design [157] if we treat the doses as experimental arms. Other methodologists have also recently addressed the topic of broad seamless dose-finding trials [158]. These proposals are obviously ambitious, covering the combined goals of trials at phases I, II and III. However, they illustrate how dose-finding could be embedded into an entire drug development program to maximise the chances of identifying the best dose for patients.

Justifiable operating characteristics and statistical error rates will naturally vary with the incidence of the disease. Logically, overall probabilities of failing to stop in genuinely toxic or ineffective scenarios should be similar to type I error rates typically used in trials, and the probabilities of correctly identifying one of the most superior doses should be comparable to conventional power.

Without doubt, this would require larger dose-finding trials. However, our proposals could be viewed as reallocating some patients from late phase trials to early phase stages of seamless trials, so that total sample sizes are not necessarily increased. Relating to this theme, [154] extolled that a dose-finding trial should not be seen as a challenge to be overcome as quickly as possible, but as an opportunity to optimise the way that an experimental treatments is given and maximise benefit for patients.

Regarding trial reporting, we advise that investigators identify the assumptions implicit in their design and justify them with reference to the nature of the treatment. We recommend that outcomes are always reported broken down by the doses investigated. A goal of dose-finding trials is to evaluate outcomes under a set of investigational doses and pooling outcomes across doses obfuscates that goal. Furthermore, investigators should discuss how the data concur or refute the assumptions, and the implications of violated assumptions on conclusions and recommendations.

## Conclusion

We conclude that dose-escalation clinical trials routinely use methods whose assumptions are violated by the outcomes observed. Specifically, methods that implicitly assume that efficacy monotonically increases in dose are very commonly used. However, we have demonstrated that the probabilities of disease response frequently do not increase with dose. We have shown this across a range of treatment types, including monotherapies and combinations. Consequently, dose-finding trials risk recommending unjustifiably high doses that may be harmful to patients. We recommend that trialists consider experimental designs that allow toxicity and efficacy outcomes to jointly determine the doses given to patients and recommended for further study.

## Supplementary Information

**Additional file 1** Supplementary material.

## Data Availability

The datasets generated and/or analysed during the current study are available in the UBIRA eData repository, 10.25500/edata.bham.00000337 R code to reproduce all statistics, tables and figures is freely available at https://github.com/brockk/dosefindingdata

## Abbreviations

A+B designs: A family of rule-based phase I clinical trial designs
AML: Acute myeloid leukaemia
BOIN: Bayesian Optimal Interval design, a phase I clinical trial design
CLL: Chronic lymphocytic leukaemia CR: Complete response
CRM: Continual Reassessment Method, a phase I clinical trial design
DLT: Dose limiting toxicity
IQR: Inter-quartile range
iwCLL: International Workshop in Chronic Lymphocytic Leukaemia
NSCLC: Non-small-cell lung cancer
MTD: Maximum tolerable dose
OR: Objective response
OS: Overall survival
PD: Progressive disease
PD-1: Programmed cell death protein 1
PFS: Progression-free survival
PK/PD: Pharmacokinetics / pharmacodynamics
PR: Partial response
RECIST: Response Evaluation Criteria in Solid Tumours
RP2D: Recommended phase 2 dose
SD: Stable disease
