# Supplementary material for: Is more better? An analysis of toxicity and response outcomes from dose-finding clinical trials in cancer
Source: BMC Cancer. 2021 Jul 5;21:777. doi: 10.1186/s12885-021-08440-0 (PMC8256624; doi:10.1186/s12885-021-08440-0)
Supplement: Supplementary file 1 — Additional file 1 Supplementary material. [file 12885_2021_8440_MOESM1_ESM.pdf]

Supplementary material to Is more better? An analysis of toxicity and response outcomes from dose-finding clinical trials in cancer

Kristian Brock[1], Victoria Homer[1], Gurjinder Soul[2], Claire Potter[1], Cody Chiuzan[3], and Shing Lee[3]

[1]Cancer Research UK Clinical Trials Unit, University of Birmingham, UK

[2]Institute of Cancer and Genomic Sciences, University of Birmingham, UK

[3]Mailman School of Public Health, Columbia University, New York, NY, USA

November 2020

1 Supplementary Methods

1.1 Graphical illustration of curve height

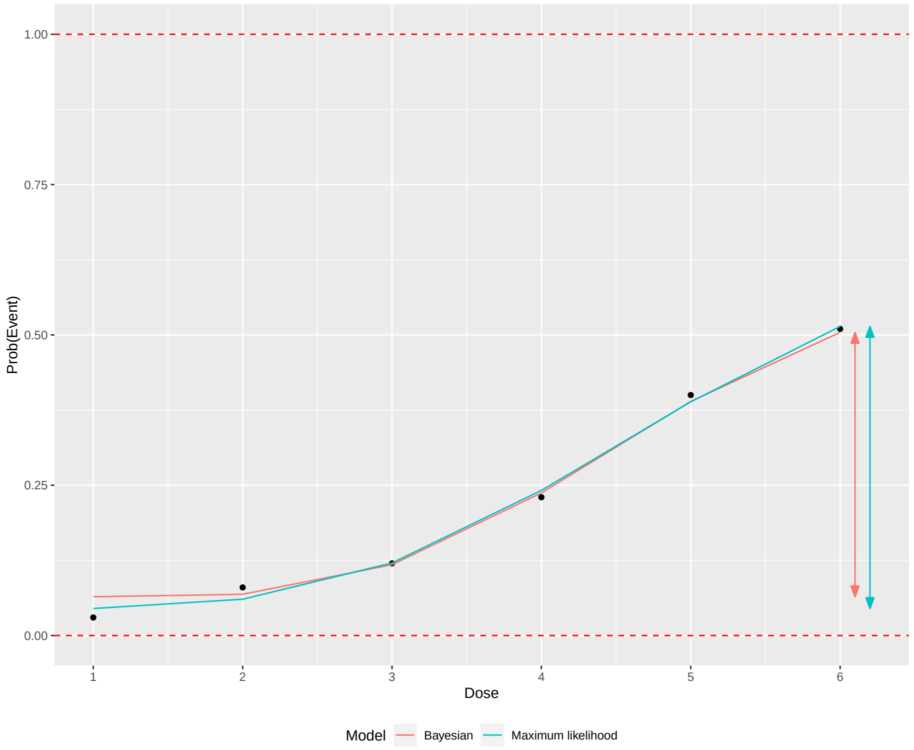

Figure 1: Visual demonstration of fitted Emax curves and the concept of curve height. $n = 100$ binary event outcomes were simulated at each of six dose-levels with event probabilities $(0.01, 0.07, 0.12, 0.25, 0.47, 0.52)$. The heights of the fitted curves are demonstrated by the vertical lines with arrows on the right-hand side, measuring the distance from the fitted curve at the lowest dose to the highest dose. A modest amount of difference is visible between the lines fit by maximum likelihood and Bayesian models.

1.2 Prior distributions

The canonical form for the Emax model is

$$y_i = E_0 + \frac{D_i^N \times E_{max}}{D_i^N + ED_{50}^N} \tag{1}$$

where

- y_i is the event probability under the dose given to patient i;
- D_i is the dose given to patient i;
- E_0 is the event probability when exposure is zero, or the zero-dose effect;
- E_{max} is the maximum effect attributable to dosing, i.e. the height of the sigmoidal curve;
- ED_{50} is the dose that produces half of E_{max};
- $N > 0$ is the slope factor, determining the steepness of the dose-event curve.

This parameterisation, however, presents difficulties when specifying priors for a binary response. For a binary outcome, the response variable is a probability and is thus defined only on the closed interval $[0, 1]$. Under the parameterisation above, we require that $E_0 \in [0, 1]$ and $E_0 + E_{max} \in [0, 1]$. However, it is difficult to specify a prior on the sum of two parameters. It is much easier to respecify the Emax model as:

$$y_i = \frac{\gamma + (ED_{50}/D_i)^N \times E_0}{1 + (ED_{50}/D_i)^N} \tag{2}$$

where

- $\gamma = E_0 + E_{max}$ is the maximum event probability;

- and all other variables retain their previous interpretation.

It is now simple to ensure that $E_0 \in [0, 1]$ and $\gamma \in [0, 1]$ using priors. Our research question involves investigating the values estimated for E_0 and γ in different clinical trials. The event rates that manifest depend on the activity of the treatment and doses selected for investigation. A completely inert treatment could produce event rates of zero. Excessively low doses of a treatment that otherwise has the potential to be active could also produce event rates of zero. Likewise, particularly active or toxic therapies could produce very high event rates, even at the lower doses of those under investigation. Each of these phenomena is plausible for therapies at the beginning of their clinical research pathway like those investigated in dose-finding trials. Thus, it is appropriate to place uniform priors on E_0 and γ over the interval $[0, 1]$.

We use folded-normal priors on ED_{50} and N with hard lower bounds at 0 because these parameters cannot take negative values, and no upper bounds.

In a study of J doses, we anticipate that ED_{50} takes the value $J/2$, i.e. we expect that the centre dose-level produces half of the maximum effect attributable to dosing. Naturally, however, we acknowledge that there is considerable uncertainty on the location of ED_{50} and set the prior standard deviation of ED_{50} to be $J - 1$. These hyperparameters ensure that the majority of the probability mass covers the doses under investigation whilst allocating approximately 25% of the mass to doses that exceed the maximum dose under investigation.

We use a folded-normal prior on N with hard lower bound at 0 and no upper bound. Greater values for N reflect steeper dose-event curves. The value $N = 1$ corresponds to a special null case of the Emax model called the hyperbolic model [1]. We assume a prior mean of 1 for N. Again, we acknowledge the material uncertainty in this variable, selecting a prior standard deviation of 3. After allowing for the lower bound, this yields a 90% prior credible interval for N of

(0.25, 6.56).

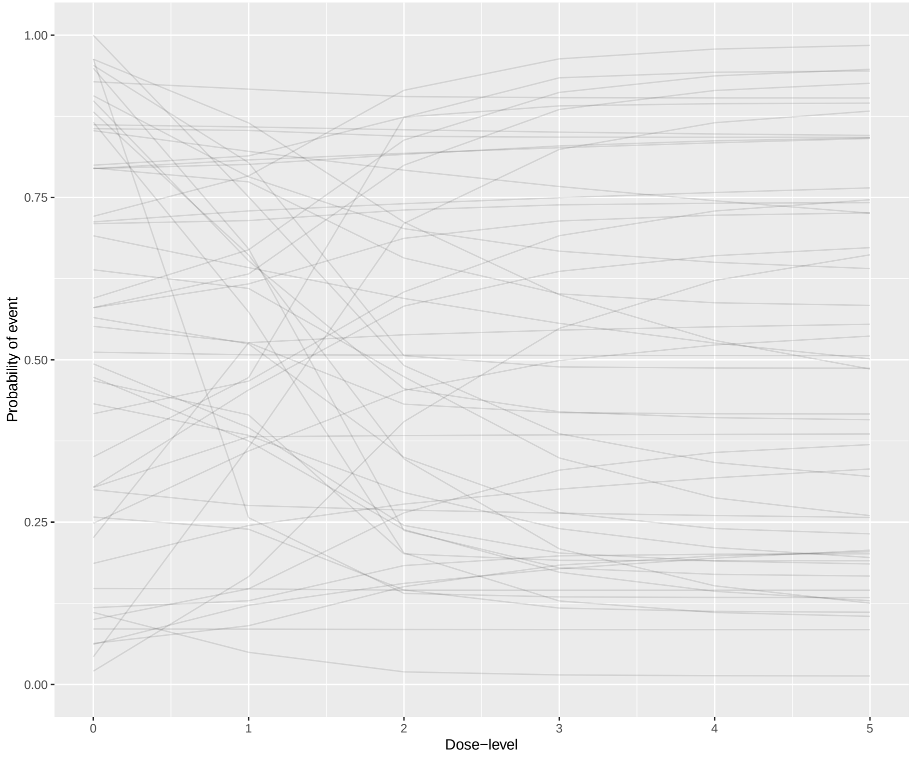

Figure 2: Prior predictive dose-event curves. A candidate value for each of the four Emax parameters was sampled from each of the described prior distributions. The fitted values determined by these parameter values were then calculated using the Emax model with $D = 0, 1, 2, 3, 4, 5$ to produce a single fitted curve. This process was then repeated fifty times. The same priors were used for both DLT and OR outcomes.

Figure 2 shows 50 dose-event curves sampled from the prior predictive distribution in a scenario with five doses. These were constructed by sampling values for the parameters from the prior distributions described above and then plugging the values into (2) with $D_i = 0, 1, ..., 5$. In this manner, a single set of

parameter values produces one dose-event curve. The process was then repeated 50 times.

In this modestly-sized sample, we see dose-event curves that start low and then increase, as we might expect to uncover for dose-toxicity curves in cytotoxic treatments. We see curves that start low and stay low, that could be consistent with inert treatments. We also see curves that start high and stay high, consistent with treatments where all the investigative doses are in the active or toxic range. Finally, we see some curves that start high and then decrease. We might not expect to see many of these in reality but it is preferable that their existence is admitted by our parameter prior choices. Furthermore, curves can plateau at any event probability between 0 and 1. Our prior parameters generate data that are consistent with our broad range of expectations of dose-event curves.

1.3 Appraising Bayesian model fits

For all Bayesian models, we recorded the number of divergent transitions, the number of times the maximum tree-depth was exceeded, and the number of Monte Carlo chains with low Bayesian fraction of missing information (BFMI). The presence of each of these features can signal poor quality sampling that does not reflect the true posterior distribution.

2 Supplementary Results

2.1 Curve heights by disease

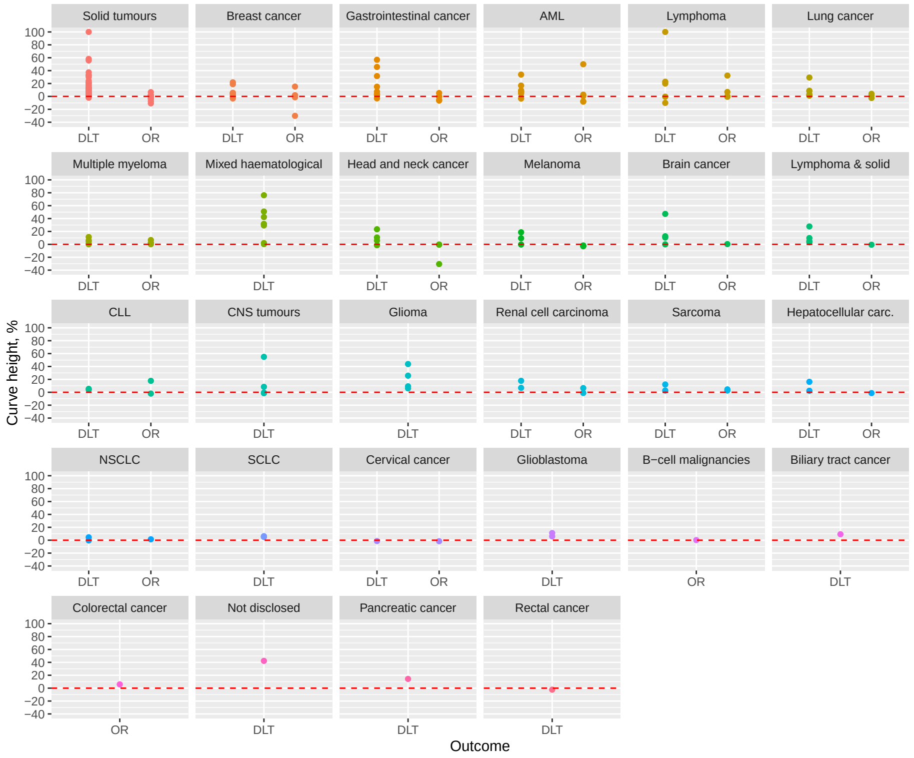

Figure 3: Heights of dose-DLT and dose-OR curves by type of disease. The dashed red lines reflect a curve height of zero where there is no association between dose and event. Positive values reflect that event probabilities increase as dose is increased, and negative values reflect a decreasing probability as dose is increased.

2.2 Curve heights by experimental design

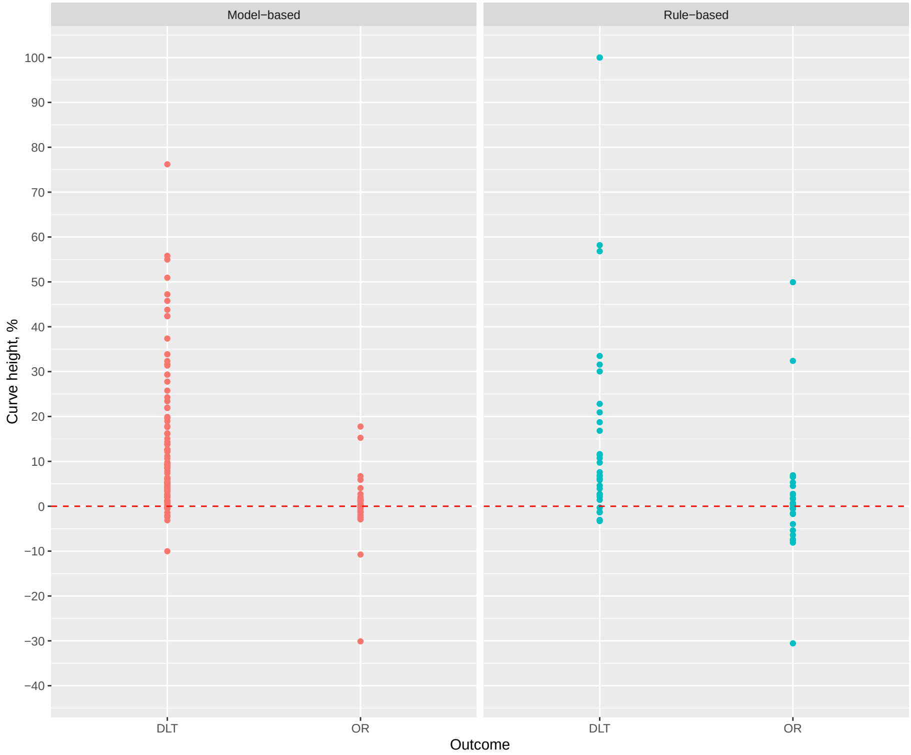

Figure 4: Heights of dose-DLT and dose-OR curves by class of experimental dose-finding design. The dashed red lines reflect a curve height of zero where there is no association between dose and event. Positive values reflect that event probabilities increase as dose is increased, and negative values reflect a decreasing probability as dose is increased.

2.3 Fitted series and curve heights by maximum likelihood models

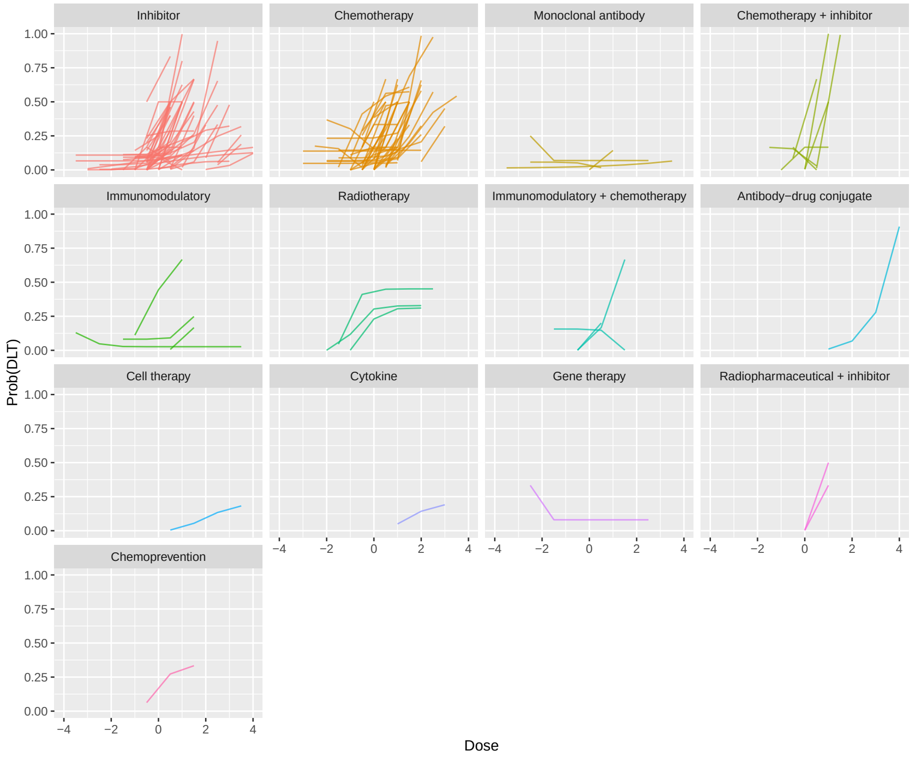

Figure 5: Fitted dose-DLT curves yielded by maximum likelihood models. For presentation, doses are centralised at zero (i.e. the average dose-level for each series is subtracted) and up to the middle nine doses are shown, to allow the series to be visualised together.

.

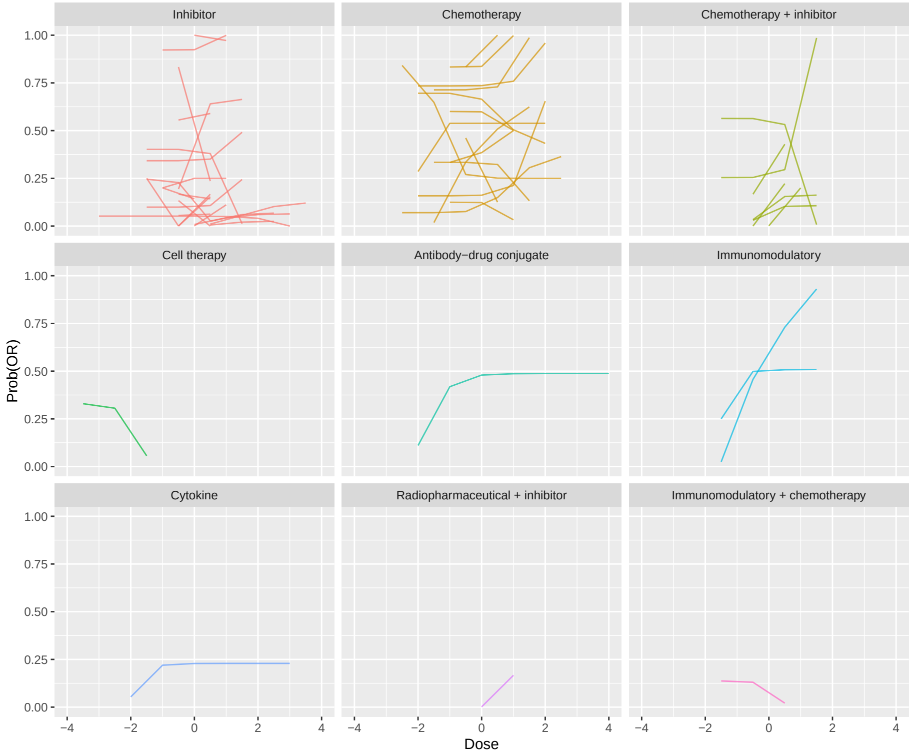

Figure 6: Fitted dose-OR curves yielded by maximum likelihood models. For presentation, doses are centralised at zero (i.e. the average dose-level for each series is subtracted) and up to the middle nine doses are shown, to allow the series to be visualised together.

.

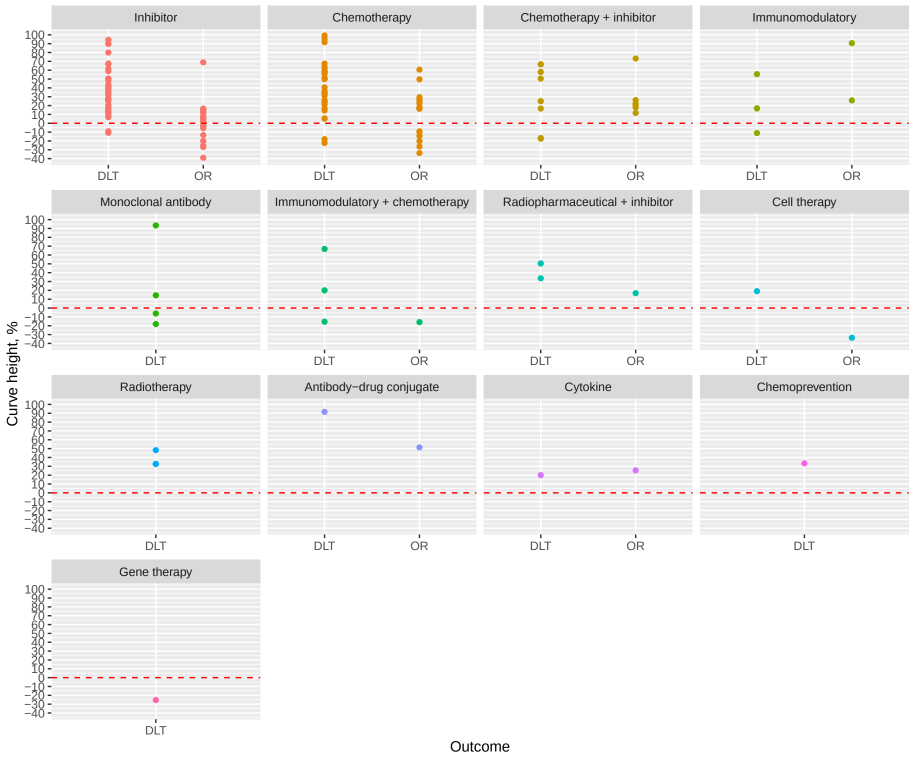

Figure 7: Heights of dose-DLT and dose-OR curves estimated by maximum-likelihood models. The dashed red lines reflect a curve height of zero where there is no association between dose and event. Positive values reflect that event probabilities increase as dose is increased, and negative values reflect a decreasing probability as dose is increased.

2.4 Bayesian model fit diagnostics

None of the fitted Bayesian model fits exceeded the maximum tree-depth in sampling, or suffered from low BFMI. Seven of the DLT models and seven of the OR models had at least one divergent transition amongst the 4000 samples sought from the joint posterior distribution in each instance. Twelve of these fourteen had fewer than seven divergent transitions. Inferences from all fourteen

instances were checked by plotting the sampled dose-event curves alongside the observed trial outcomes to ensure that the posterior inferences were a faithful representation of the data in each case.

References

[1] J. Macdougall, "Analysis of Dose–Response Studies—Emax Model," in *Dose Finding in Drug Development*, Springer New York, 2006.
